# Yellow Mealworm Protein for Food Purposes - Extraction and Functional Properties

**DOI:** 10.1371/journal.pone.0147791

**Published:** 2016-02-03

**Authors:** Xue Zhao, José Luis Vázquez-Gutiérrez, Daniel P. Johansson, Rikard Landberg, Maud Langton

**Affiliations:** Department of Food Science, Swedish University of Agricultural Sciences, Uppsala, Sweden; Agricultural University of Athens, GREECE

## Abstract

A protocol for extraction of yellow mealworm larvae proteins was established, conditions were evaluated and the resulting protein extract was characterised. The freeze-dried yellow mealworm larvae contained around 33% fat, 51% crude protein and 43% true protein on a dry matter basis. The true protein content of the protein extract was about 75%, with an extraction rate of 70% under optimised extraction conditions using 0.25 M NaOH, a NaOH solution:ethanol defatted worm ratio of 15:1 mL/g, 40°C for 1 h and extraction twice. The protein extract was a good source of essential amino acids. The lowest protein solubility in distilled water solution was found between pH 4 and 5, and increased with either increasing or decreasing pH. Lower solubility was observed in 0.5 M NaCl solution compared with distilled water. The rheological tests indicated that temperature, sample concentration, addition of salt and enzyme, incubation time and pH alterations influenced the elastic modulus of yellow mealworm protein extract (YMPE). These results demonstrate that the functional properties of YMPE can be modified for different food applications.

## Introduction

In view of the increasing world population and consumer demand for protein, sustainable protein production with low environmental impacts will be a serious future challenge. It has been suggested that edible insects can be a source of protein, since they are efficient feed converters with high nutritional value [1]. Consumption of insects is practised in many regions, but intact insects or whole insect food products have low acceptance for human consumption in most countries in the Western world. However, proteins extracted from insects and used as a pure ingredient for different food applications might have greater success in terms of acceptance as a human food.

Yellow mealworm (*Tenebrio molitor*), an edible insect, is gaining attention as a source of protein for food purposes worldwide [1]. Fresh yellow mealworm larvae contain about 15% fat and 20% protein [2,3]. A number of studies have also investigated their content of minerals, vitamins, amino acids and fatty acids [2–4]. Several methods for oil and protein extraction from mealworm larvae [4], honey bees [5], soybeans [6], ground maize [7] and cottonseed [8] have been reported, but the quality of the extracts is affected by the extraction procedure. Oil can be extracted by organic solvents or physical expelling, but safety and environmental and health issues have increased concern regarding industrial processes [9]. Ethanol is a safe organic solvent and has been investigated for defatting soybeans [6], ground maize [7] and *Quercus suber* L. fruits for human food purposes [9]. Ethanol is also suitable as an extraction solvent for defatting food materials to be used for further protein extractions.

In order to use protein from yellow mealworm larvae for human foods in a cost-effective way, protein extraction yield needs to be optimised and protein purity and functionality need to be characterised. Therefore, further research is needed to identify and refine processing parameters that affect the functionality and quality of the protein. Amino acid composition, water and fat/oil absorption capacity, protein solubility, microstructure of the yellow mealworm protein extract (YMPE) dispersion and rheological properties are important attributes for the use of YMPE as a food ingredient.

The aim of this study was to extract proteins from yellow mealworm larvae for use as a food ingredient and to determine physicochemical and functional properties relevant for food applications of the proteins extracted.

## Materials and Methods

### Mealworms, chemicals and reagents

Freeze-dried yellow mealworm (*Tenebrio molitor*) larvae were purchased from the commercial supplier Firma Cricket Express, Bohus, Gothenburg, Sweden. The worms were grown on organic feed which mainly consisted of wheat, wheat bran and carrot. The larvae were not starved before being killed. NaCl, NaOH, Na_2_SO_4_ and HCl, hexane and isopropanol were purchased from VWR (Darmstadt, Germany) and ethanol from Solveco Chemicals AB (Malmö, Sweden). Bradford reagent and bovine serum albumin (BSA) were obtained from Sigma-Aldrich Co. (St. Louis, MO, USA). Transglutaminate was kindly provided by Mühlenchemie GmbH & Co. (Ahrensburg, Germany). All reagents and chemicals were of analytical grade.

### Proximate analysis

Around 1 kg of freeze-dried worms was ground using a food processor (Braun Combimax 600) for 0.5–1 minute. Approximately 3 g of ground worms were dried at 105°C overnight for calculation of dry matter (DM) and moisture content [10]. Ash was determined by incineration at 550°C for 3 h using the basic AOAC method [10]. Crude protein (CP) content was determined at the Department of Animal Nutrition and Management (DANM), SLU, with the Kjeldahl method (Kjeltec System 2460 Distilling Unit) using a protein-to-nitrogen conversion factor of 6.25 [10]. Fat content was determined using the method from [11]. Afterwards, the solvent was removed at 40°C under reduced pressure using a rotary evaporator. All determinations were performed in triplicate. The results were expressed on both a sample weight and dry matter basis.

### Defatting of yellow mealworms

Defatting of yellow mealworm larvae was evaluated by testing the effects of solvent to material ratio (5 mL/g), temperature (40°C), time (60 min *vs*. overnight = ~18 h) and extraction twice on the yield of fat in duplicate samples. The protein content of defatted worms was also investigated. Ethanol (99.5%) was used as the extraction solvent. The defatting conditions were set according to the literature [6,7,9]. The amount of extracted fat was determined gravimetrically and the yield was expressed on a wet weight basis.

### Optimisation of protein extraction conditions

The ethanol-defatted mealworm product was used for protein extraction by NaOH. A fractional factorial design (two levels, four factors) was used to evaluate the impact of four variables (factors) simultaneously in a minimum number of trials according to the experimental design. The four factors were: NaOH concentration (0.1 M *vs*. 0.5 M), ratio of NaOH solution to defatted worm (6:1 mL/g *vs*. 20:1 mL/g), temperature (20°C and 80°C) and time (30 min and 120 min). The extract yield (%) was expressed on a wet weight basis. Significant main effects and interactions on protein extraction yield were analysed using Minitab software (Minitab Inc., State College, PA, USA).

For the experiments, 1 g of ethanol defatted worm powder was dispersed in alkaline solution in plastic tubes in the designated conditions. During the extraction period, the tubes were vortexed every 15 min. The slurry was then centrifuged at 4°C for 20 min at 3500 g. The supernatant and gel layer were removed and a second extraction was carried out on the pellet. The supernatant and gel layer from both extractions were collected together and the pH was adjusted to 4.3~4.5 with 2 M HCl at room temperature, followed by centrifugation at 4°C for 15 min at 2500 g. The precipitate was washed with distilled water and centrifuged twice at 4°C for 10 min at 2500 g. The washed precipitate was frozen at -20°C overnight (about 18 h). The frozen precipitate was freeze-dried to obtain a final protein extract with moisture content less than 5%.

After all the experiments using the fractional factorial design had been carried out, an additional experiment with the conditions: 0.25 M NaOH solution, 15:1 mL/g NaOH solution:defatted worm ratio, 40°C and 60 min was performed, in an attempt to determine the near-optimum extraction conditions. The extraction yield and crude and true protein (TP) content were evaluated. True protein content as defined by Licitra et al. (1996) [12] was determined using the Kjeldahl method (Kjeltec System 2460 Distilling Unit) after protein precipitation with trichloroacetic acid (TCA) [12], at DANM, SLU. The extraction yield (%) and the protein extraction rate were calculated as follows:

$$Extraction\;yield\;(\%)=100\times \frac{Extract}{Sample}$$

$$Extraction\;rate\;of\;protein\;(\%)=\frac{Protein\;content\;in\;extract}{Protein\;content\;in\;sample}\times Extraction\;yield\;(\%)$$

### Batch and scale-up experiments

Batch and scale-up experiments were based on parameters identified as near-optimum during the defatting and optimisation of protein extraction experiments. Three different amounts (2 g, 10 g and 30 g) of ethanol-defatted mealworm from three defatting batch experiments were studied to examine the repeatability and scalability of protein extraction.

### Fatty acid (FA) composition of extracted fat from mealworms using ethanol

Extracted fat was combined into one sample. The fatty acids (FAs) in the fat sample were transformed to fatty acid methyl esters (FAME) in methanol in the presence of NaOH and BF_3_ as catalysts and analysed by gas chromatography [13]. The peaks were identified by comparing their retention times with that of a fatty acid mixture standard (GLC-68, Nu-Check, Elysian, MN, USA). Experiments were carried out in duplicate. The FA composition was expressed as percentage of total fatty acids.

### Quality and characteristics of YMPE

The mealworm protein extracts were combined as one sample for analysis of amino acid composition, water and fat absorption capacity, protein solubility, microstructure and rheological behaviour.

**Amino acid composition** was analysed by UHPLC using UV detection at 260 nm after hydrolysis and derivatisation at DANM, SLU, according to the procedures described by [14] and [15]. Tryptophan was not included in the analysis.

**Water absorption capacity (WAC)** was estimated according to the procedure of [6]. The YMPE (1 g) was dispersed in distilled water (10 mL), stirred for 5 min and then centrifuged at low speed (2060 g for 10 min; Sorvall Super T_21_ ST-H750 centrifuge). The released water was carefully transferred to a graduated measuring cylinder and the volume was recorded. WAC was calculated as the amount of water retained by 1 g YMPE. The experiment was performed in triplicate.

**Fat absorption capacity (FAC)** was estimated using the method described by [16]. The YMPE (0.3 g) was mixed with rapeseed oil (3 mL) in a pre-weighed 15-mL graduated centrifuged tube for 5 min. After centrifugation at 2060 g for 30 min (Sorvall Super T_21_ ST-H750 centrifuge), the supernatant was discarded and the tubes were weighed. FAC was expressed as the amount of oil retained by 1 g of YMPE. The experiment was performed in triplicate.

#### Protein solubility

The YMPE (0.5 g, 10% suspension, w/v) was dispersed in distilled water or 0.5 M (~3%) NaCl solution with pH adjusted to 3, 4, 5, 7, and 9 (by adding either 1 M HCl or 2.5 M NaOH). The dispersion was stirred for 30 min and centrifuged at 2060 g for 30 min (Sorvall Super T_21_ ST-H750 centrifuge). The supernatant was measured for protein content using the method of [17] with Bovine Serum Albumin (BSA) as standard. The experiment was performed in duplicate.

Protein solubility index (PSI) was calculated as:

$$PSI\;(\%)=100\times \frac{Soluble\;protein}{Total\;protein}$$

#### Microstructure and colour

Dispersions (3 mL) of 2% YMPE in distilled water were adjusted to pH 7, 9, 11 and 13 using 1 M NaOH during a period of 30 minutes at room temperature while stirring at 1000 r/min. For each sample, one drop (8 μL) was taken for microstructural analysis using a Nikon Eclipse Ni-U light microscope coupled to a DS-Fi2 camera (Nikon, Tokyo, Japan). The rest of the dispersion was used for colour measurements using a handheld chroma meter CM 600d KONICA MINOLTA (illuminant D65, 10° observer). A CFI Plan Fluor 20X objective and differential interference contrast (DIC) were used to obtain a good overview and comparison of the solubility of the extract at different pH levels. The colour measurement was applied to give three-dimensional colour coordinates of the dispersions. Under the same conditions, 10% YMPE was prepared and the pH was adjusted to either 7, 11 or first to pH 11 and then adjusted back to pH 7 (pH 11–7) using 1 M NaOH and 1 M HCl. For these three dispersions, one drop (8 μL) was taken for microstructural analysis and the supernatant of the rest of the dispersions was analysed for soluble protein content according to the procedures described.

#### Rheological properties

Dispersions were prepared using YMPE with distilled water or NaCl solution to achieve sample dispersion at the designated concentration (w/v). The slurry was stirred for about 30 minutes at room temperature and the pH was adjusted to either 7, 9, 11 or first to pH 11 and then adjusted back to pH 7 (pH 11–7) using 1 M NaOH and 1 M HCl. To determine the rheological properties of the YMPE slurries and gels made from these, small amplitude oscillatory shear measurements (SAOS) were performed using a C-VOR 150 rheometer (Malvern Instruments Ltd., Malvern, UK) with a DIN standard concentric cylinder system (C25, 25mm diameter inner cylinder). All samples were covered with a thin layer of paraffin oil to prevent sample evaporation. Samples were first heated from 20 to 90°C at a rate of 1°C/min (phase 1), kept at 90°C for 30 min (phase 2), cooled to 20°C at a rate of 3.5°C/min (phase 3), and kept at 20°C for 30 min (phase 4). The four-phase cycle was repeated and recorded as cycle 2. During the temperature ramp, the elastic modulus G' and phase angle were determined using a strain of 0.005, which was within the linear viscoelastic range of the samples both before and after the temperature cycles, and a frequency of 1 Hz. Following the same procedures, the rheological properties of 20% (w/v) YMPE with and without addition of 0.5% transglutaminase (TG) per gram of sample, incubated at 35.5°C for 1 h, were characterised.

### Statistical analysis

Fractional factorial experiments and their analysis were designed and performed using Minitab 16 software (Minitab Inc., State College, PA, USA). The main effects of the four factors studied and their interactions on protein extraction yield were analysed by general linear models (GLM). Results are reported as mean ± standard deviation. Data from defatting experiments, batch and scale-up experiments were evaluated by one-way analysis of variance (ANOVA) using Minitab 16. Significant differences between means were determined by the Tukey’s Multiple Comparison Test procedure. P-values <0.05 were considered statistically significant.

## Results and Discussion

### Chemical composition of freeze-dried yellow mealworm larvae

The proximate composition of freeze-dried yellow mealworm larvae in terms of dry matter, ash, fat and crude protein (CP) was determined and expressed on dry matter basis (Table 1). The content of moisture and of other components was calculated by difference.

**Table 1:** Proximate composition of yellow mealworm larvae on dry matter (DM) basis (mean ± S.D., n = 2).

Yellow mealworm larvae	Dry matter (%)	Fat (%)	Crude protein (%)	Ash (%)	Other components (%)^a^
**DM basis**	96.1 ± 0.03	32.9 ± 0.86	51.5 ± 0.51	4.9 ± 0.07	10.7 ± 0.97

^a^e.g. carbohydrates and vitamins.

The moisture content of freeze-dried yellow mealworm was around 4%, which was an advantage in the subsequent defatting process. The fat content on a DM basis was about 33%. This was slightly higher than the value (27%) reported by Yi et al. [4], but slightly lower than the value (35%) reported by [2]. The slight discrepancies were probably due to different diets being fed to the yellow mealworms and different age or size of the mealworms [18]. The CP content of yellow mealworms in our study was comparable that reported by [2] and [4]. About half the DM of yellow mealworms was CP (Table 1). As expected, the CP content (51.5% of DM) was higher than the TP content (43% of DM), which indicated that the measured CP content overestimated TP content on a dry matter weight basis (8.5%) due to some non-protein nitrogen (NPN), including chitin, present in mealworms [19,20]. Therefore, true protein content might be a better estimate of actual protein content in yellow mealworms. The larvae also contained about 10% other components, such as carbohydrates and vitamins. Most of the carbohydrates may come from food remaining in the gastrointestinal tract of the larvae, since those used in this study were not starved prior to killing. Their diet mainly consisted of wheat, wheat bran and carrot, which have a high content of carbohydrates.

As expected, yellow mealworm larvae were a good source of protein and their protein content could be high enough for industrial food production purposes. The larvae also had a high content of fat as a by-product and possibly also other components such as chitin, minerals and vitamins.

### Defatting of yellow mealworms

Defatting of yellow mealworms using different solvents showed similar defatting efficiency for ethanol and a mixture of hexane and isopropanol (HIP; 3:2 v/v) studied under the test conditions (Table 2). Extraction yield was about 30% for all treatments evaluated, with no statistically significant differences between treatments. The CP and TP content of the ethanol-defatted mealworms was around 77% and 64%, respectively (Table 2). These values were comparable to those obtained in the HIP treatment, suggesting that ethanol could be a good alternative to HIP for efficient removal of fat without lowering the protein content in defatted worms. Overnight extraction only led to slightly higher extraction yield, while being more energy- and time-consuming. Extraction for 1 h was acceptable for extracting fat at a high yield. Therefore, ethanol (99.5%) was used for defatting mealworms at a solvent to material ratio of 5 mL/g, at 40°C for 60 min and extraction twice in the subsequent experiment.

**Table 2:** Fat extraction yield from mealworms under different defatting treatments, and crude protein and true protein content of defatted mealworms^a^.

Defatting treatment	Extracted fat, % of sample	Crude protein, % of DM	True protein, % of DM
**Hexane:isopropanol (3:2, v/v) + Na**_**2**_**SO**_**4**_	31.6 ± 0.86	78.3	65.4
**Ethanol, 1 h**	33.1 ± 0.63	77.8	64.3
**Ethanol, overnight**	34.8 ± 0.29	77.4	64.5

^a^Extracted fat content values are mean ± standard deviation of duplicates; crude protein and true protein content are single measurements. There were no statistically significant differences in yield of extracted fat between the different defatting treatments.

### Optimisation of protein extraction conditions

The results of the extraction experiment performed according to the fractional factorial experimental design and the extraction yields under different conditions are shown in Table 3.

**Table 3:** Experiments using the decoded fractional factorial design.

Experimental design (Decoded matrix)							Results
Block	Std order	Run order	NaOH concentration (M)	Solution: Material (mL/g)	Temperature (°C)	Time (min)	Extraction yield (%)^a^
1	15	1	0.1	20	20	120	48.4
1	16	2	0.5	20	20	30	52.2
1	18	3	0.5	6	80	30	44.4
1	19	4	0.1	20	80	30	52.3
1	22	5	0.3	13	50	75	49.7
1	21	6	0.3	13	50	75	49.7
1	20	7	0.5	20	80	120	18.6
1	17	8	0.1	6	80	120	42.4
1	24	9	0.3	13	50	75	52.2
1	23	10	0.3	13	50	75	50.4
1	14	11	0.5	6	20	120	-
1	13	12	0.1	6	20	30	36.1
2	5	13	0.1	6	80	120	41.2
2	4	14	0.5	20	20	30	50.2
2	3	15	0.1	20	20	120	50.2
2	1	16	0.1	6	20	30	31.9
2	8	17	0.5	20	80	120	19.6
2	10	18	0.3	13	50	75	50.6
2	12	19	0.3	13	50	75	52.0
2	7	20	0.1	20	80	30	52.4
2	11	21	0.3	13	50	75	50.9
2	6	22	0.5	6	80	30	42.4
2	2	23	0.5	6	20	120	49.6
2	9	24	0.3	13	50	75	49.9

^a^The extraction yield for one of the treatments was missing due to an experimental mistake.

**Fig 1:**
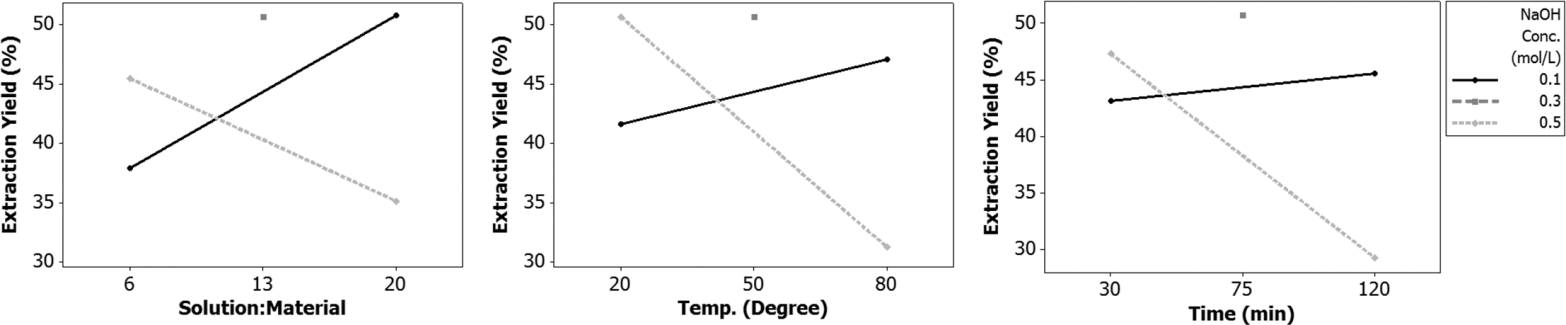
Interaction study on protein extraction yield from defatted mealworms. The factors NaOH concentration, NaOH solution-to-defatted worm ratio, temperature and time were investigated.

Main effects of the factors tested and their interactions on mealworm protein extraction yield were analysed (Fig 1). These main effects were: NaOH concentration, temperature and time, but significant interactions were also found and thus we focused on these. The interactions between the NaOH concentration and the other factors studied (NaOH solution-to-defatted worm ratio, temperature, and time) had significant effects on the yield of protein extraction (Fig 1). Low concentration of NaOH was not enough to achieve complete protein extraction and showed an upward trend as the NaOH solution-to-defatted worm ratio, extraction temperature and time increased. However, at too high NaOH concentration, part of the extracted protein was hydrolysed and the extraction yield decreased with increasing ratio, temperature and time (Fig 1).

Extraction at mid-range NaOH concentration (0.30 M), NaOH solution to defatted worm ratio (13:1), temperature (50°C) and time (75 min) gave higher yield than at other operating parameters during the experiment (Table 3 and Fig 1). However, this may be because high temperature and long extraction time can cause reduction of protein activity and thermal denaturation [8]. Moreover, the colour of the protein extract became darker with increased temperature and time. A complementary experiment with 0.25 M NaOH, NaOH solution-to-defatted worm ratio (15), temperature (40°C) and time (60 min) was carried out in an attempt to identify the near-optimum conditions and some of the corner points (Table 4). Although the protein extraction yield was not highest in these near-optimum conditions, the TP content (79%) and the extraction rate (65.1%) of TP were highest (Table 4). This indicates that the alkaline extraction method worked well when using 0.25 M NaOH, NaOH solution-to-defatted worm ratio (15), temperature (40°C) and time (60 min) for extraction twice. This optimised protein extraction method was used in further studies.

**Table 4:** Additional experiments to establish near-optimum conditions (NaOH concentration, ratio of NaOH solution-to-defatted worm, temperature, time) for protein extraction.

Operating parameters	Ext. yield	Crude protein	True protein	Ext. rate of CP	Ext. rate of TP
	(dry matter weight basis)				
**0.25 M, 15, 40°C, 60 min**	53.0	83.3	79.0	56.7	65.1
**0.30 M, 13, 50°C, 75 min**	51.6	82.3	70.8	54.6	56.8
**0.10 M, 20, 80°C, 30 min**	52.5	85.4	76.9	57.6	62.8
**0.10 M, 20, 20°C, 120 min**	46.1	82.2	76.1	48.7	54.6
**0.50 M, 6, 20°C, 120 min**	55.1	80.4	70.9	56.9	60.8
**0.50 M, 20, 20°C, 30 min**	45.2	79.4	70.2	46.1	49.3

### Batch and scale-up experiments

On a dry matter weight basis, the TP content of defatted mealworm from three batches of experiments with ethanol (99.5%), ratio of ethanol to material (5 mL/g), temperature (40°C) and time (60 min) was 61.3%, 62.0% and 61.4%, respectively. There was no significant difference in extraction yield, which ranged from 55.8% to 58.5% (Table 5). This indicates that it is possible to scale up the protein extraction at laboratory level.

**Table 5:** Extraction yield of mealworm protein after three batch defatting treatments^a^.

Defatting treatment	1	2	3
Sample amount (g)	Extraction yield (%)		
**2**	56.5 ± 0.21	57.5 ± 1.27	56.4 ± 0.28
**10**	56.1 ± 0.71	57.9 ± 0.28	57.5 ± 2.69
**30**	55.8 ± 0.07	58.5 ± 0.78	55.8 ± 0.64

^a^Values are mean ± standard deviation of duplicate measurements. There were no statistically significant differences in extraction yield.

Based on six representative runs of the experiment, the average TP content of extracts and the extraction rate of TP were calculated. On a dry matter weight basis, the TP content was 74.6±0.92% and the extraction rate was 69.9±1.40%. The average TP content of extracts in the batch and scaled-up experiments was slightly lower than the corresponding content in the protein extraction optimisation experiment (79%; Table 4), but the extraction rate of TP was higher (65.1%). This means that the protein extraction efficiency was comparable during the scaled-up experiments, although there was a slightly lower TP content in the extracts due to the lower TP content of the defatted mealworms obtained after the three defatting batch experiments.

The repeatability and scalability of defatting and protein extraction were regarded as acceptable during the batch and scale-up experiments at laboratory level. The extraction yield was higher in our study compared with previous studies [4,5]. Ozimek *et al*. obtained protein concentrate (64.2% crude protein) from honey bee by similar alkaline extraction method under somewhat different experimental conditions [5]. In the paper by Yi *et al*., three fractions were obtained from yellow mealworm by water extraction (centrifugation at 15,000 g for 30 min at 4°C): a supernatant, pellet, and residue. The protein content was 17–23%, 33–39%, 31–47% in these fractions, respectively [4]. At a laboratory level both water extraction and alkaline extraction methods appear appropriate, but at industrial level, the aqueous extraction method would be more difficult and costly to implement at such high extraction force described (15,000 g). The alkaline extraction method would be more realistic for food manufacturing conditions. It is a commonly used method for preparing protein concentrates and the nutritional quality of the honey bee protein was improved after alkaline extraction in Ozimek’s study [5]. However, the extraction method in our study has to be optimized at industrial level both for extraction yield and protein quality.

### Fatty acid composition of worm oil extracted by ethanol

The FA composition of the oil extracted using ethanol from freeze-dried yellow mealworms in this study was similar to that found for mealworm larvae by others [2,3] (Table 6). The extracted oil in our study also contained high levels of oleic (46.2%) and palmitic (23.7%) acids, and the level of oleic acid was substantially higher than that found by [2] and [3]. However, those authors reported that mealworm larvae contained high levels of linoleic acid, while the level in our study was relatively low (Table 6). This may be due to some oxidation occurring during extraction and storage. Insect meals are generally high in unsaturated FAs, which makes them susceptible to oxidation unless they are treated with an antioxidant [3]. The results in our study show the composition of fat under ethanol extraction conditions. Moreover, the FA profile in our study may to a large extent reflect the FA composition of the diet fed to the yellow mealworm larvae [21].

**Table 6:** Comparison between the fatty acid composition of oil extracted using ethanol from freeze-dried yellow mealworm larvae in this study^a^ and of fresh yellow mealworm larvae^b^ as reported [2].

Fatty acid, unit (% of total fatty acids)	Oil extracted by ethanol from freeze-dried yellow mealworm larvae^a^	Fresh yellow mealworm larvae^b^
**Capronic acid, C6:0**	1.2 ± 0.00	-
**Myristic acid, C14:0**	5.6 ± 0.14	6.4
**Palmitic acid, C16:0**	23.7 ± 0.42	28.7
**Palmitoleic acid, C16:1**	3.8 ± 0.14	6.1
**Stearic acid, C18:0**	3.9 ± 0.00	2.3
**Oleic acid, C18:1**	46.2 ± 0.21	27.7
**Linoleic acid, C18:2**	3.8 ± 0.21	23.1

^a^Fatty acids which were unidentified and present in percentages below 1% are not listed in the table. These fatty acids accounted for 11.8% of total fatty acids.

^b^The data were reported by Jones LD *et al*., 1972.

### Amino acid composition

The protein extracts prepared in our study appeared to be a good source of essential amino acids (Table 7). The extracts contained almost all the essential amino acids in quantities that are necessary for humans [22], although the content of total sulphur amino acids (methionine+cysteine: 21.3 g/kg protein) was slightly lower than the recommended level for humans (Table 7). The content of tryptophan was not determined. However, it might not be a limiting amino acid, since [4] reported that the tryptophan concentration in yellow mealworm larvae was 12 mg/g CP, twice as much as the recommended level for humans. The sum of total essential amino acids (EAA) (449.3 g/kg protein) in our study was sufficient to meet recommendations for humans (sum 277 g/kg protein). The amino acid pattern in our study was similar to that found by [3] and [4]. Amino acid recovery was 94% based on TP content. It is possible that untested amino acids such as tryptophan and taurine may have contributed to the total. In order to determine the quality of the protein extracts obtained in this study, more information on the digestibility of protein and amino acids has to be collected in further studies. Although protein quality and digestibility of mealworms has been determined in rats [23], there is a need for corresponding data from humans.

**Table 7:** Amino acid profile of protein extracts from freeze-dried yellow mealworm larvae and the recommendation for adults [22] ^a^.

Amino acid, unit (g/kg protein)	Protein extracts from freeze-dried yellow mealworm larvae	2013 FAO^a^
**Essential amino acid (EAA)**		
**Histidine**	24.1	15
**Isoleucine**	50.7	30
**Leucine**	83.0	59
**Lysine**	59.0	45
**Methionine+Cysteine**	21.3	22
**Phenyl-alanine+tyrosine**	109.1	38
**Threonine**	36.5	23
**Tryptophan**	-	6
**Valine**	65.6	39
**Sum of EAA**	449.3	277
**Non-essential amino acid**		
**Alanine**	69.8	
**Arginine**	55.5	
**Asparagine or aspartic acid**	93.3	
**Glutamic acid or glutamine**	128.8	
**Glycine**	52.9	
**Ornithine**	1.9	
**Proline**	48.5	
**Serine**	40.2	
**Sum of total AA**	940.2	

^a^The data were reported by FAO, 2013.

### Water and fat absorption capacity

The water and fat/oil absorption capacity of the protein extract from yellow mealworm larvae was found to be 1.87±0.04 (mL/g) and 2.33±0.03 (g/g), respectively. In food applications, the water absorption capacity is related to the ability to retain water against gravity and includes bound water, hydrodynamic water, capillary water and physically entrapped water [24]. Some functional properties (solubility, foaming, emulsification, viscosity, gelation and coagulation) can be interpreted as influenced by protein-water interactions [25]. Thus, information about the water absorption capacity of the protein extract is necessary. The amount of water absorbed by proteins might be influenced by its amino acid profile, charge characteristic, conformation, hydrophobicity, pH, temperature, ionic strength and protein concentration [24]. The oil absorption capacity is mainly attributed to the physical entrapment of oil and to the number of non-polar side-chains of proteins that bind the fatty acids in the oil [26]. Therefore, it is difficult to compare the values obtained in our study with results found in other studies. Further research is needed to collect more information on the water and fat absorption capacity of the protein extracts under different processing conditions, such as heat treatment.

### Protein solubility

**Fig 2:**
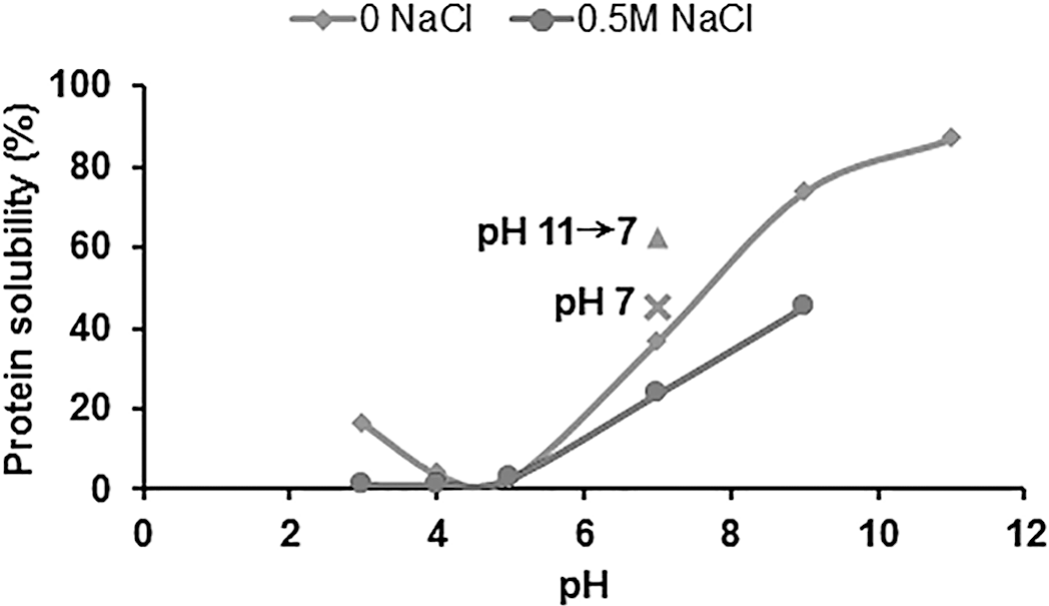
Protein solubility. The solubility profile of protein powder extracted from freeze-dried yellow mealworm larvae.

To better understand the quality of the protein extract, its solubility was evaluated (Fig 2). The protein solubility of the extract in distilled water was at a minimum between pH 4 and 5, and increased below and above this pH range, indicating that the isoelectric point is located in this range, as expected. The solubility reached its highest value at pH 9 (74%). Lower solubility was observed on adding NaCl to the solution. Similar trends were observed in aqueous and saline solution, but the protein solubility of the extract in 0.5 M NaCl solution was at a minimum between pH 3 and 4 and increased below and above this range. It has been suggested that addition of NaCl could result in a shift in the isoelectric point to a more acidic pH, which could be explained by the change in charge characteristics due to ion (Cl^-^) binding effects [27]. Data on the solubility profile of protein extracts from yellow mealworm larvae was not found in other literature. However, some studies have examined the protein solubility of lentil (*Lens culinaris Medic*) protein isolate [28] and chickpea proteins [29]. These studies have also reported lower protein solubility in NaCl solution than in water, possibly due to the salting-out effect and shifting of isoelectric point towards more acidic pH.

### Microstructure

**Fig 3:**
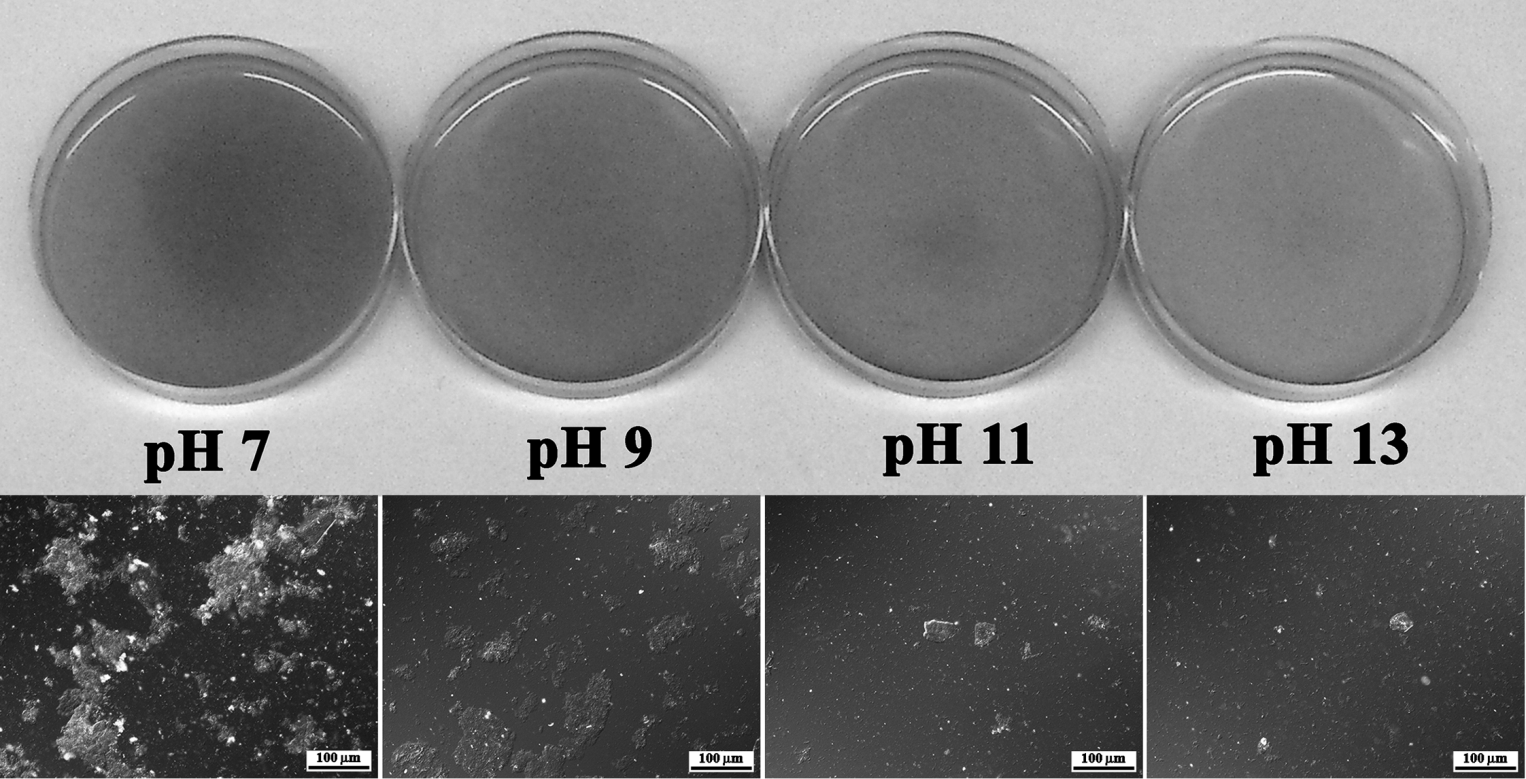
YMPE dispersions at a glance. Top row: Colour appearance of 2% dispersions (w/v) in distilled water of protein extract from freeze-dried yellow mealworm larvae at pH 7, 9, 11 and 13. Bottom row: corresponding differential interference contrast (DIC) micrographs.

Representative images of the 2% dispersions (w/v) at different pH are shown in Fig 3. With increasing pH from 7 to 13, the aggregation and particle size of proteins in the dispersions decreased due to the increased solubility of the proteins with increasing pH (Fig 3). There was also a change in appearance, with the dispersions becoming clearer, brighter and more yellow with increasing pH (Fig 3). Colour measurements confirmed this change. The L*-values indicating lightness of the dispersions from 0 (dark) to 100 (light) were 28.55, 30.18, 33.96 and 34.56 at pH 7, 9, 11 and 13, respectively. The chromaticity coordinate b* (yellow-blue direction running from +60 to -60) values was 12.09, 15.85, 16.94 and 18.99 with increasing pH from 7 to 13.

**Fig 4:**
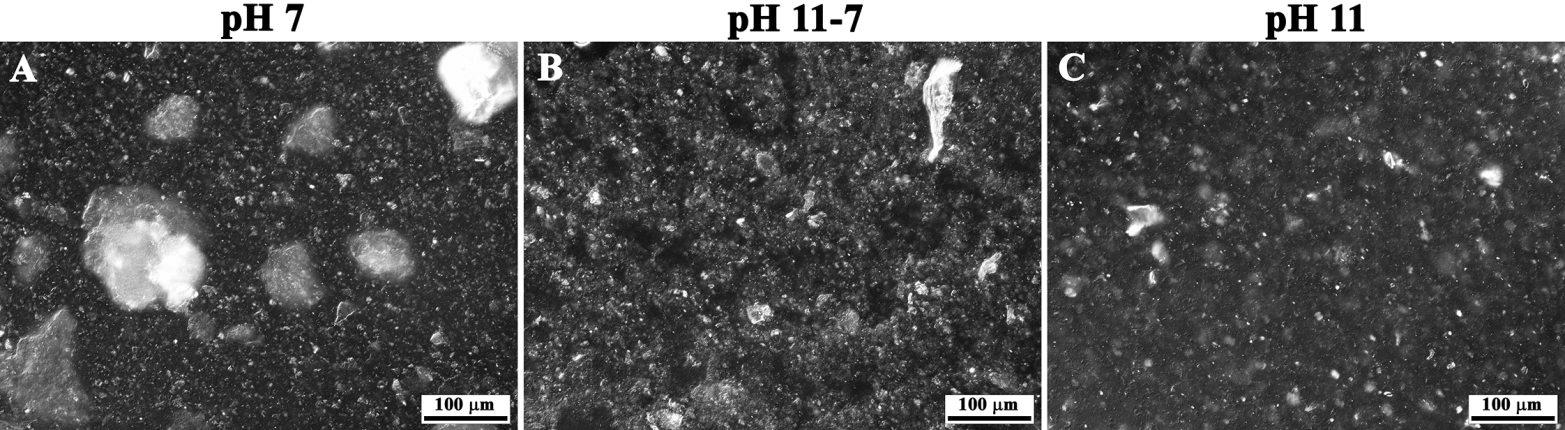
Differential interference contrast (DIC) micrographs. It shows network structure of 10% dispersions (w/v) in distilled water of protein extract from freeze-dried yellow mealworm larvae at pH 7; 7 (adjusted from 11); and 11.

Based on the results shown in Fig 3, the microstructure of 10% dispersions (w/v) in distilled water of YMPE at pH 7, pH 7 after adjustment from pH 11, and pH 11 was studied (Fig 4). The particle size in the dispersion at pH 7 (adjusted from 11) was intermediate between that in the dispersions at pH 7 (larger particle size) and pH 11 (smaller particle size). When the pH of dispersions was raised to 11, the aggregate structures opened and the protein solubility increased, resulting in fewer particles and a decrease in particle size. As pH was lowered back to 7, the dissolved proteins did not form aggregate structures of the same size, resulting in smaller protein particles compared with the sample which was not adjusted to pH 11 (Fig 4). Higher degree of aggregation and larger protein particles were observed in the dispersion at pH 7 (lowered from 11) compared with the dispersion at pH 11. This could be due to the intermediate solubility of the protein extract at pH 7 (lowered from 11), compared with at pH 7 and at pH 11 (Fig 2). This information might be useful in modifying the functionality of the protein extract for different food applications. The processability, texture, flavour and keeping qualities of food are controlled not only by chemical composition, but also by microstructure [30]. Through adjusting the microstructure, the stability, texture and sensory properties of food products could be modified for demands.

### Rheological properties of YMPE

**Fig 5:**
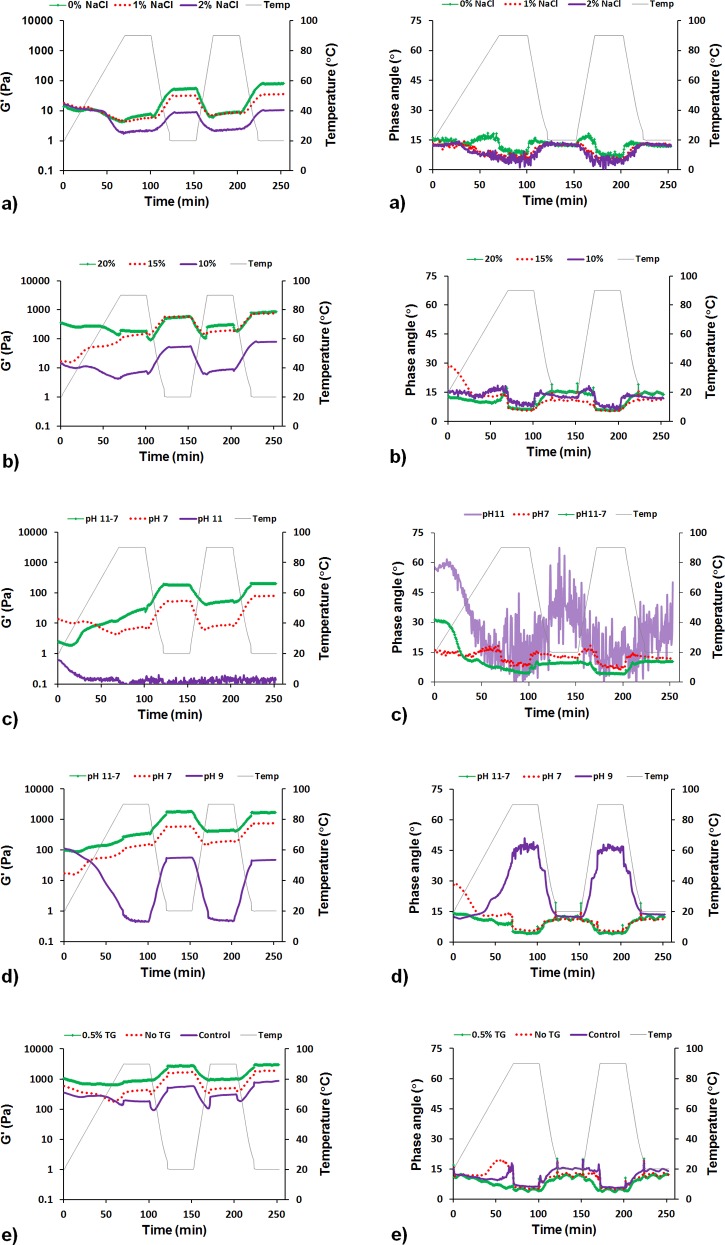
Rheological properties of YMPE. Left column: Dynamic modulus G' of YMPE during the heating and cooling phases. Right column: Corresponding phase angle data. Identical labels indicate G' and phase angle data obtained from the same experiments. a): 10% YMPE, w/v, at pH 7 with different NaCl concentrations; b): different YMPE concentrations in distilled water, w/v, at pH 7; c): 10% YMPE in distilled water, w/v, at different pH levels; d): 15% YMPE in distilled water, w/v, at different pH levels; e): 20% YMPE in distilled water, w/v, at pH 7 with and without TG after 1 h incubation at 35.5°C, and control group (no TG and no incubation).

The YMPE dispersions were prepared under various conditions. These samples were a viscous fluid in appearance, looking like smooth fruit pulps, purees or pastes such as peanut butter. Changes in the rheological properties of the dispersions during heating and cooling were investigated (Fig 5).

#### Impact of temperature on rheological properties of YMPE

Gelling behaviour (here taken as elastic modulus, G') and its temperature dependence are important for final applications of the proteins [4] and were therefore investigated. As shown in Fig 5, G' values generally showed similar trends for most samples during the heating and cooling phases between temperatures of 20 and 90°C. The G' values decreased during the heating phases and increased during the cooling phases, with slightly higher values in the second cycle. Phase angle decreased slightly with increasing temperature and increased with decreasing temperature. However, the phase angle did not increase to 45° or above, except for the sample at pH 9, where the phase angle was strongly dependent on the temperature and increased with increasing temperature. The pH 11 sample had very low G' values and a large variation in the phase angle, making it difficult to determine whether the change with temperature was due to an actual change in the sample or not. There were spikes in the phase angle when it changed from a temperature gradient to a constant temperature. This was probably mainly the result of it switching mode, although there was also a time gap which might have led to some small changes.

#### Impact of salt concentration on rheological properties of YMPE

With addition of 1% NaCl, there was a decrease in G' (Fig 5A). With 2% NaCl solution, very weak networks were obtained. This may be explained by the decreased solubility of the sample in the presence of NaCl (Fig 2).

#### Impact of sample concentrations on rheological properties of YMPE

The G' values increased with increasing sample concentration (Fig 5B). The maximum G' value obtained with 10% YMPE (80 Pa) was much lower than that obtained with 20% YMPE (870.8 Pa) or 15% YMPE (755.6 Pa), and was not high enough to form a firm structure (Fig 5B). Similar findings have been made in a study using freeze-dried supernatant fraction of proteins from mealworm [4]. Those authors found that mealworm supernatant gels at 20°C and a concentration of 15% w/v (corresponding to an actual protein concentration of 8.3%) had a G' value of around 100 Pa.

#### Impact of pH on rheological properties of YMPE

The rheological properties were strongly influenced by changes in pH. The G' values of dispersions with sample concentrations of 10% and 15% at pH 7 (lowered from 11) were 2–3 times higher than those of dispersions at pH 7 (Fig 5C and 5D). This could be due to the protein being dissolved at pH 11 and then reformed as smaller particles when the pH was lowered, leading to a general decrease in particle size (Fig 4). This change in particle size distribution may contribute to the increase in G' compared with pH 7 samples [31]. Another explanation could be that the dissolved material formed strands connecting the larger particles together, leading to an increase in G'. The G' values of the dispersions at pH 9 (0.4–111 Pa) and 11 (0.03–0.6 Pa) were both very low (Fig 5C and 5D). This is most likely due to the high solubility of the protein, resulting in fewer and smaller particles at these pH levels (Figs 3 and 4), whereas a more molecular solution needs more protein to form a continuous gel network. The sample at pH 11 did not show any distinct changes with temperature, possibly due to instrument limitations, and remained liquid at the end of the test. However, the pH 9 sample showed a marked decrease in G' accompanied by a distinct increase in phase angle with increasing temperature, indicating a change towards a very viscous gel on going from 90°C to 20°C. The higher content of particles in the 15% YMPE dispersion at pH 9 compared with the 10% YMPE dispersion at pH 11 may partly explain the differences in rheological properties. In addition, food products with pH values above 7 are rare and are also difficult to consume. The treatment at pH 7 (lowered from 11) improved the gelation capacity of YMPE and provides better chances for a future application of YMPE as a food ingredient.

#### Impact of transglutaminase (TG) on rheological properties of YMPE

As TG catalyses crosslinks between glutamine and lysine [32–35], it was expected that the stiffness of the YMPE network would increase with addition of TG. With regard to temperature dependence, the trends were similar for YMPE with and without TG (Fig 5E). However, there was a significant increase in the maximum value of G' during the temperature ramp for the 0.5% TG/sample treatment (3048 Pa) compared with sample without TG addition (1959 Pa) and the control treatment (870.8 Pa) (Fig 5E). The difference between the G' values for the TG and TG-free samples with incubation compared with no incubation treatment may be explained by very slow solubility or swelling of particles during incubation. The mealworm protein and its components were preheated or enzyme-hydrolysed during incubation, which could lead to exposure of reactive groups and enhanced potential to react with each other [33]. The difference in G' values for samples with and without TG addition after the same incubation period is most likely due to increased intra- and intermolecular crosslinking induced by the TG [32,34]. Addition of 0.5% TG to YMPE could apparently enhance gel stiffness to some extent.

## Conclusions

Freeze-dried yellow mealworm larvae were found to contain around 33% fat, 51% crude protein and 43% true protein by dry matter weight. Ethanol can be used for extraction of fat from yellow mealworm larvae. The extracted oil, rich in oleic and palmitic acids, could be used as a side product after further purification. Proteins were extracted with a 0.25 M NaOH, a NaOH solution to defatted worm ratio of 15:1 mL/g, 40°C for 60 min and extraction twice. The TP content of the protein extract was about 75% at an extraction rate of 70%. This alkaline extraction method was easy and feasible to apply, and provided acceptable extraction yield. The protein extract obtained was a good source of essential amino acids.

The lowest protein solubility was reached around isoelectric point, and increased with either increasing or decreasing pH. The protein solubility of the extract was lower in 0.5 M NaCl solution than in distilled water. Rheological tests showed that the elastic modulus was influenced by temperature, sample concentration, salt and enzyme addition, incubation and pH alteration. These results demonstrate that the functional properties of YMPE could be modified to make it suitable for different food applications.

## References

[1] Van Huis A. Potential of Insects as Food and Feed in Assuring Food Security. Annu Rev Entomol. 2013; 58(1):563–83. 23020616 10.1146/annurev-ento-120811-153704

[2] Jones LD, Cooper RW, Harding RS. Composition of Mealworm Tenebrio molitor Larvae. J Zoo Anim Med. 1972; 3(4):34–41.

[3] Finke MD. Complete nutrient composition of commercially raised invertebrates used as food for insectivores. Zoo Biol. 2002; 21(3):269–85.

[4] Yi L, Lakemond CMM, Sagis LMC, Eisner-Schadler V, van Huis A, van Boekel MAJS. Extraction and characterisation of protein fractions from five insect species. Food Chem. 2013 12 15; 141(4):3341–8. 10.1016/j.foodchem.2013.05.115 23993491

[5] Ozimek L, Sauer WC, Kozikowski V, Ryan JK, Jorgensen H, Jelen P. Nutritive value of protein extracted from honey bees. J Food Sci. 1985 9; 50(5):1327–9.

[6] L’Hocine L, Boye JI, Arcand Y. Composition and functional properties of soy protein isolates prepared using alternative defatting and extraction procedures. J Food Sci. 2006 4; 71(3):C137–45.

[7] Kwiatkowski JR, Cheryan M. Extraction of oil from ground corn using ethanol. J Am Oil Chem Soc. 2002 8 1; 79(8):825–30.

[8] Zhang BN, Cui YD, Yin GQ, Li XM, Zhou XX. Alkaline extraction method of cottonseed protein isolate. Mod Appl Sci. 2009; 3(3):77–82.

[9] Ferreira-Dias S, Valente DG, Abreu JMF. Comparison between ethanol and hexane for oil extraction from Quercus suber L. fruits. Grasas Aceites. 2003 12; 54(4):378–83.

[10] AOAC. AOAC (Association of Official Analytical Chemists), Official Methods of Analyses of Association of Analytical Chemist 15th ed. AOAC, Washington, DC.; 1990.

[11] Hara A, Radin NS. Lipid extraction of tissues with a low-toxicity solvent. Anal Biochem. 1978 10 1; 90(1):420–6. 727482 10.1016/0003-2697(78)90046-5

[12] Licitra G, Hernandez TM, Van Soest PJ. Standardization of procedures for nitrogen fractionation of ruminant feeds. Anim Feed Sci Technol. 1996 3; 57(4):347–58.

[13] Azadmard-Damirchi S, Dutta PC. Stability of minor lipid components with emphasis on phytosterols during chemical interesterification of a blend of refined olive oil and palm stearin. J Am Oil Chem Soc. 2008 1; 85(1):13–21.

[14] Liu H, Chang B, Yan H, Yu F, Liu X. Determination of Amino-Acids in Food and Feed by Derivatization with 6-Aminoquinolyl-N-Hydroxysuccinimidyl Carbamate and Reversed-Phase Liquid-Chromatographic Separation. J Aoac Int. 1995 6; 78(3):736–44.

[15] Liming W, Jinhui Z, Xiaofeng X, Yi L, Jing Z. Fast determination of 26 amino acids and their content changes in royal jelly during storage using ultra-performance liquid chromatography. J Food Compos Anal. 2009; 22(3):242–9.

[16] Lin M, Humbert E, Sosulski F. Certain Functional Properties of Sunflower Meal Products. J Food Sci. 1974; 39(2):368–70.

[17] Bradford MM. A rapid and sensitive method for the quantitation of microgram quantities of protein utilizing the principle of protein-dye binding. Anal Biochem. 1976 5 7; 72:248–54. 942051 10.1016/0003-2697(76)90527-3

[18] Finkel AJ. The lipid composition of Tenebrio Molitor larvae. Physiol Zool. 1948; 21(2):111–33. 18913652 10.1086/physzool.21.2.30151989

[19] Barker D, Fitzpatrick MP, Dierenfeld ES. Nutrient composition of selected whole invertebrates. Zoo Biol. 1998; 17(2):123–34.

[20] Finke MD. Estimate of chitin in raw whole insects. Zoo Biol. 2007; 26(2):105–15. 10.1002/zoo.20123 19360565

[21] Finke MD. Complete nutrient content of four species of commercially available feeder insects fed enhanced diets during growth. Zoo Biol. 2015 Nov-Dec; 34(6):554–64. 10.1002/zoo.21246 26366856

[22] FAO. Dietary protein quality evaluation in human nutrition In Report of an FAO Expert Consultation, (pp. 27). Auckland, New Zealand: Food and Agriculture Organization of the United Nations 2013.

[23] Goulet G, Mullier P, Sinave P, Brisson G. Nutritional evaluation of dried Tenebrio Molitor larvae in rat. Nutr Rep Int. 1978; 18(1):11–5.

[24] Naik A, Raghavendra SN, Raghavarao KSMS. Production of coconut protein powder from coconut wet processing waste and its characterization. Appl Biochem Biotechnol. 2012 7; 167(5):1290–302. 10.1007/s12010-012-9632-9 22434355

[25] Damodaran S. Food proteins and their applications, (Paraf A. ed.), Marcel Dekker, New York pp. 1–21. 1997.

[26] Al-Kahtani HA, Abou-Arab AA. Cereal Chemistry, 70, pp. 619–626. 1993.

[27] Schut J. Meat Emulsion, in: Friberg S. (Ed.) Food Emulsions. Marcel Dekker Inc, New York, pp: 385–458. 1976.

[28] Mashair A. Suliman, Abdullahi H. El Tinay, Abd Elmoneim O. Elkhalifa, Elfadil E. Babiker, Elhadi A.I. Elkhalil. Solubility as Influenced by pH and NaCl Concentaration and Functional Properties of Lentil Proteins Isolate. Pak J Nutr. 2006 6 1; 5(6):589–93.

[29] Liu L h., Hung T v. Functional Properties of Acetylated Chickpea Proteins. J Food Sci. 1998; 63(2):331–7. 10.1111/j.1750-3841.2010.01639.x 20629870

[30] Aguilera JM. Why food microstructure? J Food Eng. 2005; 67(1–2):3–11.

[31] Lopez-Sanchez P, Nijsse J, Blonk HCG, Bialek L, Schumm S, Langton M. Effect of mechanical and thermal treatments on the microstructure and rheological properties of carrot, broccoli and tomato dispersions. J Sci Food Agric. 2011; 91(2):207–17. 10.1002/jsfa.4168 20862717

[32] Ramírez-Suárez JC, Xiong YL. Effect of transglutaminase-induced cross-linking on gelation of myofibrillar/soy protein mixtures. Meat Sci. 2003 10; 65(2):899–907. 22063454 10.1016/S0309-1740(02)00297-8

[33] Gauche C, Barreto PLM, Bordignon-Luiz MT. Effect of thermal treatment on whey protein polymerization by transglutaminase: Implications for functionality in processed dairy foods. LWT—Food Sci Technol. 2010 3; 43(2):214–9.

[34] Sun XD, Arntfield SD. Gelation properties of chicken myofibrillar protein induced by transglutaminase crosslinking. J Food Eng. 2011 12; 107(2):226–33.

[35] Sun XD, Arntfield SD. Gelation properties of myofibrillar/pea protein mixtures induced by transglutaminase crosslinking. Food Hydrocoll. 2012 6; 27(2):394–400.

